# 3D Stereophotogrammetry Analysis and Psychosocial Outcomes of Injectable Fillers in Gender‐Affirming Facial Contouring: A Prospective Analysis

**DOI:** 10.1111/jocd.71130

**Published:** 2026-08-19

**Authors:** Bruna S. F. Bravo, Ana Luísa Leopoldino de Souza, Victoria Kong, Leonard Knoedler, Michael Alfertshofer, Robert Gaudin, Deise Caldas Kühlman, Juliana Martins Leal, Marina Ramos Baeta Neves, Leonardo Gonçalves Bravo, Regina Casz Schechtman, Sueli Carneiro

**Affiliations:** ^1^ Bravo Private Clinic Rio de Janeiro Brazil; ^2^ Faculty of Medicine Rio de Janeiro State University Rio de Janeiro Brazil; ^3^ Division of Plastic Surgery, Department of Surgery Yale School of Medicine New Haven Connecticut USA; ^4^ Department of Oral and Maxillofacial Surgery Charité – Universitätsmedizin Berlin, Corporate Member of Freie Universität Berlin, and Humboldt‐Universität Zu Berlin Berlin Germany; ^5^ Institute of Postgraduate Dentistry Private University in the Principality of Liechtenstein Triesen Liechtenstein; ^6^ Department of Preventive and Community Dentistry, Faculty of Dental Surgery Rio de Janeiro State University Rio de Janeiro Brazil

**Keywords:** 3D photogrammetry, dermal fillers, facial contouring, gender‐affirming care, hyaluronic acid, patient‐reported outcomes, transgender health

## Abstract

**Background:**

Gender‐affirming facial procedures alleviate dysphoria, yet prospective data on nonsurgical interventions remain scarce. Objective data on anatomical and psychosocial outcomes following injectable fillers in transgender populations are poorly defined.

**Aims:**

To quantitatively evaluate soft‐tissue changes and psychosocial outcomes following gender‐affirming facial contouring with hyaluronic acid (HA) fillers.

**Patients/Methods:**

In this prospective, single‐center study, 50 transgender patients (25 trans women and 25 trans men) underwent individualized HA filler treatments. Three‐dimensional (3D) stereophotogrammetry was performed using a VECTRA (Canfield Scientific, Parsippany, NJ, USA) to assess facial soft‐tissue changes at baseline and 30 days post‐treatment. Linear distances were measured on 3D facial images using the VECTRA Analysis Module software with integrated digital caliper tools. Psychosocial outcomes were measured using validated Likert‐type measures of satisfaction and gender identification, including the Global Aesthetic Improvement Scale (GAIS).

**Results:**

Mean injected volume was 9.8 ± 3.5 mL and was well tolerated, with no serious adverse events. Satisfaction and gender identification scores improved significantly (Δ + 1.53 and + 1.71, respectively; both *p* < 0.001). The 3D analysis demonstrated significant, directionally gender‐congruent changes. Trans women showed increases in bizygomatic width (+ 3.75 mm) and labial height (+ 1.77 mm), along with a reduction in perceived chin width (−12.94 mm). Trans men exhibited increases in bigonial width (+ 5.04 mm) and chin width (+ 4.53 mm) (all *p* < 0.001). Despite millimeter‐scale changes, these modifications were associated with substantial psychosocial improvement.

**Conclusions:**

This prospective study demonstrated measurable, gender‐congruent changes in facial morphology following injectable HA filler treatment, accompanied by improvements in patient‐reported satisfaction and gender identification. Despite similar injected volumes, anatomical effects differed between groups, reflecting strategic three‐dimensional redistribution of soft tissue rather than volume alone. These findings support nonsurgical facial contouring as an effective approach for gender affirmation.

## 1 Introduction

Gender‐affirming procedures play a critical role in reducing gender dysphoria and improving quality of life for transgender individuals [1, 2, 3, 4]. While surgical facial feminization and masculinization techniques have been well‐characterized, nonsurgical approaches using injectable fillers represent an important alternative or adjunct to surgery. Advantages of these procedures include reversibility, customizability, and minimal recovery time [5, 6, 7]. Despite growing recognition of the unique aesthetic needs of transgender patients, the literature remains limited regarding evidence‐based protocols for gender‐affirming facial contouring with dermal fillers [1, 6].

Facial gender expression is determined by distinct anatomical proportions. Ideal feminine faces are characterized by a heart‐shaped contour with prominent zygomas, a tapered jawline, fuller lips, and increased vertical facial height, whereas masculine faces exhibit a rectangular shape with a 1:1:1 ratio of bitemporal, bizygomatic, and bigonial widths, along with a more prominent jaw and chin [1]. Nonsurgical techniques can address these features through strategic filler placement for malar augmentation, lip enhancement, jawline contouring, and chin modification [5, 8]. However, most published data on facial injectables in transgender patients are derived from expert opinion or extrapolated from cisgender cosmetic procedures, with a notable absence of prospective studies evaluating objective anatomical changes and patient‐reported outcomes [6].

Facial interventions for gender affirmation have been shown to significantly improve psychosocial functioning, reduce anxiety, depression, and social isolation, and enhance overall mental health and satisfaction with appearance [9, 10, 11]. These benefits underscore the medical necessity of facial gender‐affirming interventions [2]. Yet, access to such procedures remains limited, and many transgender individuals report high levels of facial dysphoria with low rates of treatment [12, 13].

Hyaluronic acid (HA) dermal fillers are commonly used to refine facial features, including in younger individuals [14]. In the United States, HA filler injections rank as the second most frequently performed nonsurgical aesthetic procedure, with approximately 1.5 million treatments carried out each year [14, 15, 16]. Owing to their consistent, reliable outcomes and their ability to address a wide range of aesthetic concerns, HA fillers have become a preferred option in many clinical settings [17, 18].

Three‐dimensional (3D) stereophotogrammetry offers objective, reproducible quantification of facial changes with a high degree of measurement precision [19, 20, 21]. This facilitates detection of subtle changes that may not be otherwise captured through conventional clinical assessment [19]. As such, it serves as a bridge between objective structural changes and subjective aesthetic perception.

The present study prospectively evaluates the anatomical and psychosocial outcomes of gender‐affirming facial contouring using injectable HA fillers in a cohort of 50 transgender patients (25 trans women and 25 trans men). The null hypothesis was that filler‐based facial contouring would not result in measurable changes in facial dimensions consistent with feminization or masculinization goals, nor would it significantly improve patient satisfaction or gender identification scores. To our current knowledge, this is the largest prospective study to date evaluating both objective facial measurements and validated patient‐reported outcomes following nonsurgical gender‐affirming facial procedures.

### 1.1 Clinical Relevance

Beyond addressing this critical gap in the literature, the findings of this study have direct clinical implications. By providing objective, quantitative evidence of gender‐congruent facial changes alongside validated psychosocial improvements, this work contributes to the development of evidence‐based frameworks for nonsurgical gender‐affirming care. Such data may assist clinicians in refining treatment planning, guiding patient counseling, and setting realistic expectations. Moreover, the integration of 3D stereophotogrammetry with patient‐reported outcomes offers a reproducible approach for evaluating treatment efficacy, thereby supporting the standardization of injectable techniques within multidisciplinary gender‐affirming care pathways.

## 2 Materials and Methods

### 2.1 Study Design and Patient Population

This prospective, single‐center study included 50 transgender patients seeking gender‐affirming facial contouring, recruited from the postgraduate institute at the Institutional Research Ethics Committee of Hospital Universitário Pedro Ernesto (HUPE), Universidade do Estado do Rio de Janeiro (UERJ) (Approval No. 7.039.521; CAAE: 82497324.7.0000.5). The sample comprised 25 trans women and 25 trans men. Participants were categorized as trans women (assigned male at birth) and trans men (assigned female at birth) based on self‐identified gender.

Inclusion criteria consisted of patients 18 years of age or older who identified as transgender and desired nonsurgical facial contouring. Exclusion criteria included a history of any prior facial surgery, receipt of any facial aesthetic treatment within the past 12 months, and receipt of any permanent facial fillers or implants. Demographic and baseline clinical characteristics were recorded. These included age, weight, height, smoking status, and history of gender‐affirming hormonal therapy.

The study protocol was approved by the Institutional Research Ethics Committee of Hospital Universitário Pedro Ernesto (HUPE), Universidade do Estado do Rio de Janeiro (UERJ) (Approval No. 7.039.521; CAAE: 82497324.7.0000.5259). All participants provided written informed consent prior to participation. Data collection and analysis were conducted in accordance with the ethical principles outlined in the Declaration of Helsinki.

### 2.2 Sample Size Calculation

The sample size calculation was performed using G*Power 3.1 (Heinrich‐Heine‐Universität Düsseldorf, Düsseldorf, Germany) for within‐subject paired comparisons. The sample size calculation was based on the primary anatomical outcome, defined as the detection of a clinically relevant change in facial linear measurements. Patient‐reported satisfaction and gender identification were considered secondary outcomes.

Based on a minimum clinically relevant difference of 2.3 mm and a standard deviation of 2.97 mm [22, 23], the standardized effect size was estimated at approximately 0.7. Assuming a two‐tailed alpha of 0.05% and 90% power, the minimum required sample was 25 participants. To allow subgroup evaluation according to gender identity, 50 patients were included in the present study, comprising 25 trans women and 25 trans men.

### 2.3 Treatment Protocol

Gender‐affirming facial contouring was performed using injectable HA dermal fillers, Rennova (Rennova, São Paulo, SP, Brazil). The choice of filler volume, injection plane, and injection technique was tailored according to each patient's gender‐affirming treatment goals. Filler was delivered via a needle for the mental region. All other regions were injected via a cannula. The plane of injection was standardized according to the target anatomical region to optimize safety and aesthetic projection (Table 1). Total injected volumes were recorded for all facial regions, including the chin, jaw, zygoma, lips, pyriform fossa, nose, nasolabial fold, malar, under‐eye, forehead, labiomandibular fold, temple, and acne scars.

**TABLE 1:** Standardized injection planes and corresponding facial regions.

Injection plane	Region
Supraperiosteal	Mental, zygomatic, and nasal (including suprachondral placement)
Subcutaneous	Mandibular
Submuscular	Periocular, lips, frontal, and malar
Subfascial	Temporal

Although filler was not injected into all evaluated regions in every patient, all facial areas were analyzed to assess indirect contour changes and overall harmonization of facial proportions following gender‐affirming treatment.

### 2.4 Outcome Measures

**FIGURE 1:**
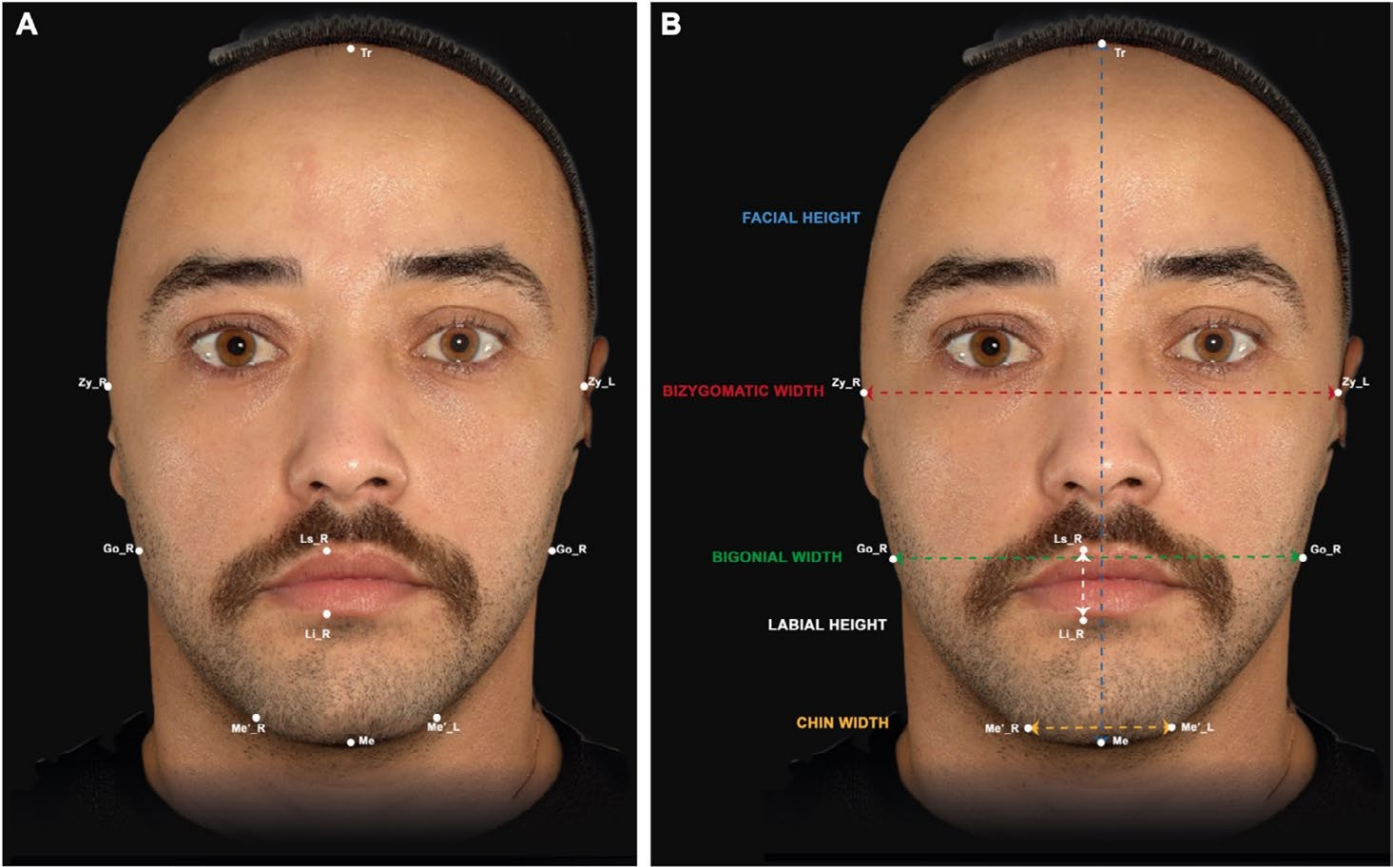
(A) Standardized anatomical landmarks used for linear measurements on the three‐dimensional (3D) facial surface. (B) Standardized digital measurements used for soft‐tissue change analysis. Illustration of key facial dimensions obtained via 3D stereophotogrammetry using VECTRA. Facial height was measured from the trichion (Tr) to the mentum (Me) (blue). Bizygomatic width represents the maximum horizontal distance between the zygions (Zy_R and Zy_L) (red). Bigonial width was defined as the distance between the gonial angles (Go_R and Go_L) (green). Labial height was measured from the crista philtri to the lower vermilion border (Ls_R and Li_R) (white). Chin width was defined as the horizontal distance across the soft‐tissue mentum (Me’_R and Me’_L) (yellow). All measurements were performed using standardized anatomical landmarks.

Patients were evaluated pre‐treatment (day 0) and post‐treatment at a 30‐day follow‐up. Outcomes were assessed quantitatively and categorized into patient‐reported psychosocial measures and objective anatomical parameters. Quantitative facial changes were evaluated using a VECTRA 3D stereophotogrammetric imaging system (Canfield Scientific, Parsippany, NJ, USA) with the VECTRA Analysis Module software. All three‐dimensional facial images were acquired using a standardized imaging protocol, with patients positioned in a natural head position in the same room under identical lighting conditions at both time points. Anatomical measurements were performed by a single blinded investigator using digital calipers on 3D models based on standardized anthropometric landmarks. The measurement protocol involved the identification of predefined facial anatomical landmarks (Figure 1A), followed by linear measurements obtained using digital calipers on the 3D models (Figure 1B).

Vertical facial height was defined as the linear distance from the trichion (Tr) to the mentum (Me). The bizygomatic width was defined as the maximum horizontal distance between the most lateral points of the zygions (Zy_R and Zy_L). Bigonial width was defined as the horizontal distance between the most lateral points of the mandibular angles (Go_R and Go_L). Chin width was defined as the horizontal distance between the most lateral points of the soft‐tissue mentum (Me’_R and Me’_L). The labial height was defined as the vertical distance from the right cristae philtrum (Ls_R) to the lower vermilion border (Li_R).

Moreover, patient satisfaction was assessed using a 5‐point Likert‐type scale (1 = very dissatisfied, 5 = very satisfied). Perceived gender congruence was assessed using a 5‐point Likert‐type scale, in which participants rated the extent to which their facial appearance aligned with their gender identity (1 = not at all aligned, 5 = completely aligned) [24]. Overall aesthetic improvement was assessed 30 days after treatment using the Global Aesthetic Improvement Scale (GAIS). Patient‐reported outcomes were evaluated using the Subject Global Aesthetic Improvement Scale (SGAIS), while investigator‐reported outcomes were assessed using the Physician Global Aesthetic Improvement Scale (PGAIS). Both scales are 5‐point ordinal measures ranging from 1 (very much improved) to 5 (worse) [25].

Participant blinding was not feasible due to the nature of the intervention. To minimize measurement bias, all 3D images were anonymized and assigned coded identifiers prior to analysis. The evaluator was blinded to both patient identity and time point, such that baseline and post‐treatment images could not be distinguished during measurement. All assessments were performed in randomized order under standardized conditions.

### 2.5 Statistical Analysis

Data were collected at baseline (Day 0) and post‐treatment (Day 30). Normality of continuous variables was assessed using the Shapiro–Wilk test. As several variables did not meet the assumption of normality, non‐parametric tests were applied for inferential analyses. Categorical variables are presented as frequencies and percentages (n, %), whereas continuous variables and patient‐reported outcome measures are presented as mean ± standard deviation (SD) for descriptive purposes and to facilitate interpretation and comparison with previous studies.

Between‐group (female vs. male) comparisons used the Mann–Whitney *U* test (continuous) or Fisher's exact test (categorical). Pre–post changes were assessed using the Wilcoxon signed‐rank test (paired), applied to questionnaire scores (satisfaction and gender identification, 1–5 scale) and facial measurements (vertical height, bigonial width, chin width, bizygomatic width, labial height). Sex‐stratified analyses compared the magnitude of change between groups using the Mann–Whitney *U* test. A two‐tailed *p* < 0.05 was considered statistically significant; significant values are highlighted in tables. Analyses were conducted in Python (version 3.12; Python Software Foundation, Wilmington, DE, USA) with SciPy (version 1.11) and pandas (version 2.1). Missing data were handled by complete‐case analysis; no imputation was performed. In addition to hypothesis testing, standardized effect sizes (Cohen's *d*) were calculated for paired comparisons to quantify the magnitude of the observed changes. Effect sizes were interpreted according to conventional thresholds as small (0.20), medium (0.50), and large (0.80) [26].

Intraobserver reliability was assessed using the intraclass correlation coefficient (ICC). Ten randomly selected patients were remeasured by the same examiner after a 2‐week interval. Reliability was evaluated using a two‐way mixed‐effects model with absolute agreement. Because this was an exploratory study evaluating multiple predefined outcomes, no formal adjustment for multiple comparisons was performed.

## 3 Results

The cohort comprised 50 transgender patients (25 trans women, 25 trans men), with a mean age of 32.2 ± 7.4 years (range 21–55), mean weight of 76.2 ± 15.2 kg, and mean height of 1.7 ± 0.1 m. HIV‐positive status was reported in 2 patients (4%), alcohol use in 18 (49%), and any smoking in 15 (33%). No statistically significant difference was observed between trans women and trans men with respect to age (*p* = 0.268). However, significant differences were found for weight (*p* = 0.018) and height (*p* < 0.001).

Mean total injected volume was 9.8 ± 3.5 mL overall (females: 10.3 ± 3.8 mL vs. males: 9.0 ± 2.9 mL; *p* = 0.384). Baseline satisfaction and gender identification scores were similar between groups (2.8 ± 0.9 and 2.8 ± 1.0, respectively), with no significant between‐sex difference (both *p* > 0.05). At the time of the procedure, 37 patients (74%) were on active hormonal therapy (trans women: 15/25 [60%]; trans men: 22/25 [88%]), 6 (12%) had previously used hormonal therapy but had since discontinued it, and 7 (14%) reported no history of hormonal therapy use. (Table 2).

**TABLE 2:** Patient characteristics.

Variable	All (*n* = 50)	Trans women (*n* = 25)	Trans men (*n* = 25)	*p*
Demographics				
Age (years)	32.2 ± 7.4	33.4 ± 9.0	31.0 ± 5.2	0.268
Weight (kg)	76.2 ± 15.2	81.0 ± 15.3	71.4 ± 13.8	0.018**
Height (m)	1.7 ± 0.1	1.74 ± 0.07	1.65 ± 0.07	< 0.001*
Clinical characteristics				
HIV‐positive	2 (4%)	2 (8%)	0 (0%)	0.490
Alcohol use	18 (49%)	10 (48%)	8 (50%)	> 0.99
Smoker (any)	15 (33%)	6 (27%)	9 (38%)	0.539
Hormonal therapy				0.021**
Currently on HT	37 (74%)	15 (60%)	22 (88%)	
Previously on HT (discontinued)	6 (12%)	6 (24%)	0 (0%)	
Never on HT	7 (14%)	4 (16%)	3 (12%)	
Treatment volume				
Total volume injected (mL)	9.8 ± 3.5	10.3 ± 3.8	9.0 ± 2.9	0.384
Session 1 volume (mL)	8.7 ± 3.0	8.1 ± 2.6	9.3 ± 3.2	0.202
Baseline pre‐treatment scores (scale 1–5)				
Satisfaction score (pre)	2.8 ± 0.9	2.6 ± 1.0	3.1 ± 0.8	0.100
Gender identification score (pre)	2.8 ± 1.0	2.6 ± 1.0	2.9 ± 1.1	0.307
Filler volume by facial area, *n* (%) treated [mean mL per treated patient]				
Chin	45 (90%) [2.7 mL]	24 (96%) [2.5 mL]	21 (84%) [2.9 mL]	0.344
Jaw	25 (50%) [6.9 mL]	1 (4%) [2.0 mL]	24 (96%) [7.1 mL]	< 0.001*
Zygoma	22 (44%) [2.8 mL]	22 (88%) [2.8 mL]	0 (0%) [−]	< 0.001*
Lips	22 (44%) [1.5 mL]	21 (84%) [1.5 mL]	1 (4%) [1.0 mL]	< 0.001*
Pyriform fossa	14 (28%) [1.0 mL]	14 (56%) [1.0 mL]	0 (0%) [−]	< 0.001*
Nose	13 (26%) [1.4 mL]	13 (52%) [1.4 mL]	0 (0%) [−]	< 0.001*
Nasolabial fold	13 (26%) [1.1 mL]	11 (44%) [1.1 mL]	2 (8%) [1.2 mL]	0.008**
Malar	12 (24%) [1.6 mL]	11 (44%) [1.6 mL]	1 (4%) [1.0 mL]	0.004**
Under‐eye	12 (24%) [0.9 mL]	10 (40%) [0.9 mL]	2 (8%) [0.9 mL]	0.021**
Forehead	5 (10%) [1.1 mL]	5 (20%) [1.1 mL]	0 (0%) [−]	0.047
Labiomandibular fold	5 (10%) [1.3 mL]	3 (12%) [1.2 mL]	2 (8%) [1.5 mL]	> 0.99
Temple	4 (8%) [2.4 mL]	4 (16%) [2.4 mL]	0 (0%) [−]	0.109
Acne scar	1 (2%) [1.6 mL]	1 (4%) [1.6 mL]	0 (0%) [−]	> 0.99

Abbreviations: HT, hormonal therapy; SD, standard deviation; Smoker variable excludes social/occasional smokers from binary categorization.

* Statistically significant difference (*p* < 0.001).

** Statistically significant difference (*p* < 0.05).

Intraobserver reliability was good to excellent across all variables. ICC values were 0.941 for vertical facial height, 0.963 for bigonial width, 0.889 for chin width, 0.927 for bizygomatic width, and 0.951 for labial height.

Regarding the distribution of filler volume across facial regions, the chin was the most commonly treated area overall (*n* = 45/50, 90%), followed by the jaw (*n* = 25, 50%), zygoma (*n* = 22, 44%), lips (*n* = 22, 44%), nose (*n* = 13, 26%), nasolabial fold (*n* = 13, 26%), pyriform fossa (*n* = 14, 28%), malar region (*n* = 12, 24%), and under‐eye area (*n* = 12, 24%). Less frequently treated areas included the temple (*n* = 4, 8%), forehead (*n* = 5, 10%), labiomandibular fold (*n* = 5, 10%), and acne scars (*n* = 1, 2%).

The treatment pattern differed markedly between groups: in trans women, the most common sites were the zygoma (22/25), lips (21/25), and chin (24/25), with a mean volume of 2.8 mL, 1.5 mL, and 2.5 mL respectively among those treated. In trans men, injections were concentrated in the chin (21/25, mean 2.9 mL) and jaw (24/25, mean 7.1 mL), reflecting the goal of masculinizing the lower face through volumization. (Table 2).

All five facial measurements changed significantly from baseline. Vertical facial height increased by a mean of +1.75 mm (193.29 to 195.03 mm; *p* < 0.001), bizygomatic width increased by +1.98 mm (145.83 to 147.81 mm; *p* < 0.001), and bigonial width increased by +2.49 mm (129.42 to 131.91 mm; *p* < 0.001). Labial height increased by +0.89 mm (22.14 to 23.02 mm; *p* < 0.001). Chin width changed significantly in both groups but in opposite directions: trans women showed a mean decrease of 12.94 mm (*p* < 0.001), consistent with facial feminization goals, while trans men showed a mean increase of 4.53 mm (*p* < 0.001), consistent with masculinization. The between‐group difference in change was highly significant (*p* < 0.001) (Table 3).

**TABLE 3:** Before vs. after treatment comparisons.

Variable	Pre‐treatment mean ± SD	Post‐treatment mean ± SD	Δ (post − pre)	*p*	Effect size	*n*
Facial Measurements (mm)						
Vertical facial height	193.29 ± 10.75	195.03 ± 11.95	+1.75	< 0.001*	0.352	50
Bigonial width (jaw)	129.42 ± 9.36	131.91 ± 8.55	+2.49	< 0.001*	−1.948	50
Chin width	45.26 ± 6.08	41.05 ± 11.63	−4.20	0.005**	−1.168	50
Bizygomatic width (cheekbones)	145.83 ± 8.58	147.81 ± 9.91	+1.98	< 0.001*	−0.329	50
Labial height (upper lip)	22.14 ± 4.22	23.02 ± 4.31	+0.89	< 0.001*	0.067	50

*Note:* Wilcoxon signed‐rank test for pre‐ and post‐treatment comparisons. Sample size (*n*) reported in the final column. Effect sizes are presented as Cohen's *d*.

Abbreviations: Δ, mean difference (post‐treatment—pre‐treatment); SD, standard deviation.

* Statistically significant difference (*p* < 0.001).

** Statistically significant difference (*p* < 0.05).

Treatment was associated with significant improvements in patient‐reported outcomes. Satisfaction scores increased from 2.84 ± 0.94 at baseline to 4.37 ± 0.78 post‐treatment (Δ = +1.53; *p* < 0.001), and gender identification scores improved from 2.78 ± 1.05 to 4.49 ± 0.65 (Δ = +1.71; *p* < 0.001) (Table 4).

**TABLE 4:** Patient‐centered outcomes.

Variable	Pre‐treatment mean ± SD	Post‐treatment mean ± SD	Δ (post − pre)	*p*	Effect size	*n*
Patient‐reported outcomes (scale 1–5)						
Satisfaction score	2.84 ± 0.94	4.37 ± 0.78	+1.53	< 0.001*	−1.608	49
Gender identification score	2.78 ± 1.05	4.49 ± 0.65	+1.71	< 0.001*	−1.768	49

	Day 30 mean ± SD					*n*
Global aesthetic improvement scale (GAIS; scale 1–5)						
SGAIS D30	1.56 ± 0.73					50
PGAIS D30	2.42 ± 0.78					50

*Note:* Wilcoxon signed‐rank test was used for pre‐ and post‐treatment comparisons. GAIS outcomes were assessed only at Day 30 and are presented descriptively. Effect sizes are presented as Cohen's *d*. Sample size (*n*) is reported in the final column.

Abbreviations: Δ, mean difference (post‐treatment – pre‐treatment); SD, standard deviation; SGAIS, subject global aesthetic improvement scale; PGAIS, physician global aesthetic improvement scale.

* Statistically significant difference (*p* < 0.001).

Overall aesthetic improvement at day 30 was favorable. The mean SGAIS score was 1.56 ± 0.73, indicating that most participants rated their appearance as much improved or very much improved following treatment. Similarly, the mean PGAIS score was 2.42 ± 0.78, reflecting a positive aesthetic improvement as assessed by the investigators (Table 4).

Standardized effect size analysis demonstrated large treatment effects for the patient‐reported outcomes, with Cohen's *d* values of 1.61 for satisfaction and 1.77 for gender identification. Subgroup analyses also demonstrated large effects for both trans women (*d* = 1.75 and 2.01, respectively) and trans men (*d* = 1.35 and 1.37, respectively) (Tables 3, 4, 5).

**TABLE 5:** Gender‐stratified treatment response: Trans women vs. trans men.

Variable	Trans women (*n* = 25)			Trans men (*n* = 25)				Between group *p*			
	Pre mean ± SD	Post mean ± SD	Δ	*p*	Effect size	Pre mean ± SD	Post mean ± SD	Δ	*p*	Effect size	
Patient‐reported outcomes (scale 1–5)											
Satisfaction score	2.60 ± 1.04	4.20 ± 0.82	+1.60	< 0.001*	−1.752	3.08 ± 0.78	4.54 ± 0.72	+1.46	< 0.001*	−1.352	0.486
Gender identification score	2.64 ± 0.99	4.36 ± 0.57	+1.72	< 0.001*	−2.005	2.92 ± 1.10	4.62 ± 0.71	+1.71	< 0.001*	−0.75	0.890
Facial measurements (mm)											
Vertical facial height	196.72 ± 9.12	199.78 ± 11.05	+3.06	< 0.001*		189.85 ± 11.31	190.28 ± 11.07	+0.43	0.300		< 0.001*
Bigonial width (jaw)	130.85 ± 9.58	130.79 ± 9.63	−0.06	0.593		127.99 ± 9.10	133.03 ± 7.33	+5.04	< 0.001*		< 0.001*
Chin width	43.86 ± 5.50	30.92 ± 4.70	−12.94	< 0.001*		46.66 ± 6.42	51.18 ± 6.32	+4.53	< 0.001*		< 0.001*
Bizygomatic width (cheekbones)	148.38 ± 7.16	152.13 ± 8.58	+3.75	< 0.001*		143.29 ± 9.25	143.49 ± 9.38	+0.20	0.180		< 0.001*
Labial height (upper lip)	23.36 ± 4.02	25.14 ± 3.35	+1.77	< 0.001*		20.91 ± 4.13	20.91 ± 4.17	+0.00	> 0.99		< 0.001*

*Note:* Δ, mean difference (post‐treatment—pre‐treatment). Between‐group p magnitude comparison of change between trans women and trans men patients. Effect sizes are presented as Cohen's *d*.

* Statistically significant difference (*p* < 0.001).

Gender‐stratified analyses revealed that improvements in satisfaction and gender identification were significant in both trans women and trans men (all within‐group *p* < 0.001), with no significant difference in the magnitude of change between groups (satisfaction: *p* = 0.486; gender identification: *p* = 0.890) (Table 5).

In contrast, the pattern of facial changes differed markedly between groups. Chin width changed significantly in both groups but in opposite directions: trans women showed a mean decrease of 12.94 mm (*p* < 0.001) while trans men showed a mean increase of 4.53 mm (*p* < 0.001), with the between‐group difference being significant (*p* < 0.001). Bigonial width increased significantly only in trans men (*p* < 0.001; trans women: *p* = 0.593), with a significant between‐group difference (*p* < 0.001). Similarly, labial height increased significantly in trans women (*p* < 0.001) but not in trans men (*p* > 0.99), and the two groups differed significantly in this change (*p* < 0.001). Vertical facial height increased significantly in trans women (*p* < 0.001) but not in trans men (*p* = 0.300), with a significant between‐group difference (*p* < 0.001). These findings reflect the distinct anatomical goals of feminizing versus masculinizing filler approaches (Table 5).

### 3.1 Harms

No serious or permanent adverse events were reported. Only mild adverse events were observed in 12 patients, including transient edema, ecchymosis, and local pain, which resolved spontaneously within 1–5 days.

## 4 Discussion

Although injectable fillers are widely used in aesthetic dermatology, there is a lack of quantitative evidence describing their role in gender‐affirming facial contouring [1, 5, 6, 7]. This prospective study integrates objective 3D stereophotogrammetric analysis with patient‐reported outcomes and demonstrates measurable, gender‐congruent changes in facial dimensions following injectable dermal filler treatment. These anatomical changes were accompanied by significant improvements in patient‐reported satisfaction and gender identification. Our findings suggest that strategically placed fillers can modify facial proportions in ways that align with masculine and feminine facial ideals.

Distinct patterns of anatomical change were observed between trans women and trans men, reflecting their different structural treatment goals. In trans men, the focus was primarily on the lower face. We observed a significant +5.04 mm increase in bigonial width and a + 4.53 mm increase in chin width. Conversely, feminizing treatments for trans women emphasized midfacial and perioral augmentation. This was achieved through a + 3.75 mm increase in bizygomatic width and a + 1.77 mm increase in labial height, coupled with a dramatic 12.94 mm mean decrease in chin width. These findings align with established anthropometric descriptions of masculine and feminine facial morphology, in which masculine faces demonstrate greater lower‐face width and projection, whereas feminine faces are characterized by greater malar prominence and softer lower facial contours [1, 5, 6, 7].

Trans women demonstrated an apparent reduction in chin width despite the volumetric nature of HA fillers. Chin width was measured directly between predefined soft‐tissue anthropometric landmarks using standardized three‐dimensional stereophotogrammetry, and intraobserver reliability for this measurement was good (ICC = 0.889). Therefore, the observed change is unlikely to be explained solely by measurement variability. Rather than representing a true reduction in tissue volume, this finding likely reflects changes in three‐dimensional facial geometry [5]. Strategic filler placement within the chin, prejowl sulcus, mental crease, and submental regions increases anterior projection while simultaneously reshaping the lower facial contour [1, 27, 28]. Consequently, the most lateral soft‐tissue landmarks may shift medially on the facial surface, producing a narrower transverse linear measurement despite overall volumization. Although standardized head positioning and blinded measurements were used to minimize methodological bias, this explanation remains hypothetical, and future studies incorporating three‐dimensional volumetric surface analyses may further clarify the mechanisms underlying this observation.

Of note, the magnitude of the observed changes was relatively small, occurring at the millimeter scale. Nevertheless, the substantial improvement in patient‐reported satisfaction (mean score increasing from 2.84 to 4.37; Δ = +1.53) suggests that these subtle volumetric redistributions were clinically perceptible and meaningful to patients. This clinical impact is supported by Wang et al., who reported that the untrained human eye is sensitive to minute facial asymmetries and deviations [29]. The eyelids were the most sensitive feature of asymmetry detection (2 mm), followed by oral commissures (3.5 mm), brow position (3.5 mm), and nasal tip and chin (4 and 6 mm, respectively). Given that most of the measured changes meet or exceeded these thresholds established by Wang et al., these findings further lend evidence to demonstrate that the success of gender‐affirming facial treatment does not require massive volumetric shifts.

Although the observed anatomical changes were relatively small in absolute terms, they exceeded the measurement variability demonstrated by the good‐to‐excellent intraobserver reliability of the stereophotogrammetric protocol (ICC 0.889–0.963). Furthermore, several changes approached or exceeded perceptual thresholds previously reported for facial asymmetry detection [30]. However, minimal clinically important differences have not yet been established for three‐dimensional anthropometric outcomes following gender‐affirming facial contouring with injectable fillers. Consequently, clinical significance should be interpreted in the context of objective anatomical changes, patient‐reported outcomes, and the absence of established MCID values for this field.

Alternatively, it remains possible that the high satisfaction scores are driven not only by these perceived visual changes but also by the procedural and expectation‐related effects inherent in gender‐affirming care [31, 32, 33]. Aesthetic interventions are known to confer benefits through mechanisms including enhanced self‐perception, expectation fulfillment, and observer‐reported improvements in social attributes [34]. The short follow‐up interval limits assessment of sustained postprocedural satisfaction. Controlled or sham‐comparator designs would help delineate these effects.

Significant improvements in gender identification were observed, with scores rising from 2.78 to 4.49 (+1.71). Of note, there was no significant difference in the magnitude of these psychosocial improvements between trans women and trans men. This suggests that the psychological benefit may be shared across the gender spectrum when aesthetic goals are met. Prior studies of gender‐affirming facial surgery have demonstrated significant improvements in psychosocial functioning, including reductions in anxiety, depression, and social distress associated with facial gender dysphoria [2, 9, 11, 35, 36]. Our findings suggest that nonsurgical interventions may provide similar psychosocial benefits for selected patients, particularly those seeking incremental changes or those who may not be candidates for surgical procedures.

Our data highlight specific clinical priorities for this patient population. The chin was the most frequently treated area (90%), followed by the jaw (50%) and zygoma (44%). These patterns mirror surgical approaches to facial gender affirmation, in which genioplasty, mandibular contouring, and malar augmentation are commonly performed procedures [37, 38]. However, unlike surgical interventions, injectable fillers offer the advantages of reversibility, minimal downtime, and the ability to titrate aesthetic changes gradually over time [5, 7, 39, 40]. These findings support the use of injectable fillers as a flexible and scalable intervention within the spectrum of gender‐affirming facial treatments. For some patients, fillers may serve as an entry point to facial modification, while for others they may function as an adjunct to surgical intervention or as a means of refining postoperative results. This versatility is particularly relevant in settings where access to surgery is limited.

At the time of treatment, approximately three quarters of patients in this cohort were on active hormonal therapy. Given that hormones induce gradual soft‐tissue and fat redistribution, our results suggest that fillers may serve as a complementary modality [41, 42, 43]. Fillers provide immediate structural contouring whereas hormonal therapy induces longer‐term soft‐tissue remodeling [43, 44]. Future studies examining the interaction between hormone therapy and filler‐based contouring may help clarify optimal timing and treatment strategies. The stability of these results at 30 days provides a promising baseline for the efficacy of nonsurgical gender‐affirming care.

## 5 Limitations

Several limitations of this study should be acknowledged. First, the absence of a control group precludes definitive attribution of the observed psychosocial improvements solely to the intervention, as concurrent factors such as expectation effects, placebo responses, or ongoing hormonal therapy may have contributed.

The follow‐up period was limited to 30 days, restricting assessment to early post‐treatment outcomes and filler integration. As HA fillers undergo gradual resorption and gender‐affirming care represents a long‐term therapeutic process, this study cannot determine the durability of anatomical changes, optimal maintenance intervals, the persistence of psychosocial improvements, or patients' adaptation to their facial changes over time [44]. Longer‐term prospective studies are necessary to characterize these outcomes.

Although 3D stereophotogrammetry provides high‐resolution and reproducible measurements, a true 3D analysis could not be performed. This is because accurate superimposition of pre‐ and post‐treatment images remains challenging in facial soft‐tissue analysis due to the absence of stable reference structures for reliable registration. As a result, linear, landmark‐based measurements were used as a pragmatic and reproducible alternative. Fourth, while validated Likert scales were used to assess satisfaction and gender identification, these measures remain subjective and may be influenced by psychological and contextual factors. The study did not incorporate broader quality‐of‐life instruments or mental health assessments, which could provide a more comprehensive evaluation of psychosocial impact.

Moreover, all procedures were performed by a single experienced injector using one commercially available hyaluronic acid filler, which may limit the applicability of the findings to other clinical settings, injector experience levels, or filler formulations.

Finally, the heterogeneity in treatment patterns, including individualized filler volumes, injection sites, and treatment strategies, reflects real‐world clinical practice but introduces variability that limits reproducibility and precludes attribution of specific anatomical changes to individual injection techniques or facial regions. Future studies using standardized treatment protocols or stratified analyses according to injection patterns are needed to better characterize technique‐specific outcomes.

## 6 Conclusion

This prospective study demonstrated measurable, gender‐congruent changes in facial morphology following injectable hyaluronic acid filler treatment, accompanied by improvements in patient‐reported satisfaction and gender identification. Despite comparable filler distribution across groups, anatomical outcomes differed according to gender identity. Trans women patients demonstrated increased midfacial projection with greater bizygomatic width, associated with mean injected volumes of approximately 1.3–1.6 mL in the malar/zygomatic regions. In contrast, trans men patients showed enhanced lower facial width, particularly at the mandibular angle, corresponding to mean volumes of approximately 1.3–1.6 mL in the jawline/gonial region. Notably, similar chin volumes produced opposite effects, with a reduction in perceived chin width in trans women patients and an increase in trans men patients. These findings highlight that outcomes depend not only on volume but on strategic three‐dimensional redistribution of soft tissue. Because this was an uncontrolled pre‐post study, improvements in patient‐reported outcomes should be interpreted with appropriate caution, and causal relationships cannot be definitively established. The relatively small sample size, short follow‐up period, and recruitment from a single Brazilian center further limit the generalizability of the findings. Although the cohort included participants with diverse racial backgrounds, all subjects were treated within one national setting, and cultural perceptions of facial attractiveness and facial anthropometry may differ across populations. Future prospective studies including comparator groups, larger and more diverse cohorts, and longer follow‐up are needed to evaluate the durability of anatomical and psychosocial outcomes and to optimize treatment protocols.

## Author Contributions

Bruna S. F. Bravo, Regina Casz Schechtman, Robert Gaudin, and Sueli Carneiro contributed to the conception and design of the study. Bruna S. F. Bravo, Ana Luísa Leopoldino de Souza, Juliana Martins Leal, Marina Ramos Baeta Neves, Leonardo Gonçalves Bravo, and Deise Caldas Kühlman were involved in data collection and patient assessment. Victoria Kong, Leonard Knoedler, Michael Alfertshofer, and Juliana Martins Leal contributed to data analysis and interpretation. Bruna S. F. Bravo, Victoria Kong, Leonard Knoedler, and Michael Alfertshofer drafted the manuscript. All authors critically revised the manuscript, approved the final version, and agreed to be accountable for all aspects of the work.

## Funding

The authors have nothing to report.

## Consent

IRB protocol ID: 7.039.521, Hospital Universitário Pedro Ernesto and 82497324.7.0000.5259, Universidade do Estado do Rio de Janeiro.

## Conflicts of Interest

Bruna S. F. Bravo is a medical consultant for Rennova, Croma Pharma, Allergan Aesthetics, Galderma Aesthetics, Merz Aesthetics, IPSA, L'Oréal, and Sinclair.

## Data Availability

The data that support the findings of this study are available from the corresponding author upon reasonable request.
